# Comparing the usefulness of four imaging techniques in the same gouty arthritis patient

**DOI:** 10.1111/j.1756-185X.2012.01744.x

**Published:** 2012-05-09

**Authors:** Houheng Su, Xia Li, Na Zhao, Hejian Zou, Lindi Jiang, Yi Zhou

**Affiliations:** 1 Department of Rheumatology, Qingdao Municipal HospitalQingdao; 2 Department of Rheumatology, Huashan HospitalShanghai, China; 3 Department of Radiology, Zhongshan HospitalShanghai, China

Dear Editor,

Gout is a paradoxical disease in that arthritis attacks are only intermittent, whereas the arthritis-causing uric acid is persistently in excess in the form of tophi. For each patient, the ultimate therapeutic ideal should be to completely eliminate all tophi. So far, clinicians have relied on physical examination and plain radiographs to assess the locations and sizes of gouty tophi.^1^ Published reports suggest that dual energy computed tomography (DECT), magnetic resonance imaging (MRI) and 2-fluoro-deoxy-d-glucose (FDG) positron emission tomography (PET) each offer a different perspective of gouty tophi.^2^ DECT is capable of discriminating urate from non-urate compounds.^3^,^4^ MRI is capable of delimiting the sizes of the tophi and their relationship to adjacent tissues.^5^ PET is capable of assessing the degree of inflammation.^6^ Because no study had used all four techniques concomitantly, no guidelines are available concerning their applications. In this paper, we used these four techniques to examine the same feet and knees of a patient with chronic tophaceous in gout. The results indicate two huge tophi that would have eluded detection and assessment by physical examination and plain radiographs alone in this patient.

The patient is a 62-year-old man with intermittent gouty arthritis attacks of the joints of the lower extremities for 20 years. Physical examination showed tophi-like swelling at the left big toe, and minor joint swelling of both knees. Serum uric acid was 8.6 mg/L. The diagnosis of gout was confirmed by crystal examination. Plain radiographs and 3.0T MRI followed conventional methods. DECT and FDG-PET utilized the Siemens Somatom Definition Syngo 2010A and the Siemens Biograph 16 (Knoxville, TN, USA), respectively.

**Figure 1:**
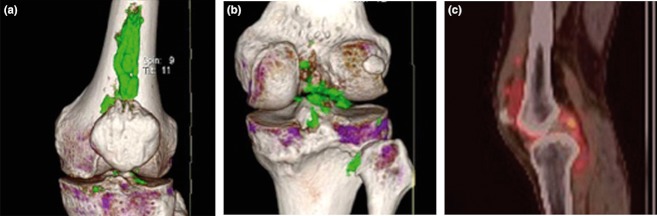
Images of the left knee. Panels (a and b) are three-dimensional reconstructed dual energy computed tomography anterior and posterior views. Green color represents urate compound. Note large compact tophus above patella in (a) and scattered green patches in (b). Panel (c) is a positron emission tomography/computed tomography side view image. Red color reflects intense 2-fluoro-deoxy-d-glucose emission.

**Figure 2:**
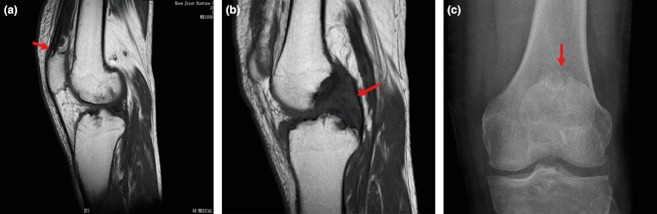
Images of left knee. Panels (a and b) are T1-weighted magnetic resonance sagittal images. Arrow in panel (a) shows bone erosion and tophus. Arrow in panel (b) shows a homongenous mass compatible with the tophus in the positron emission tomography/computed tomography scan of Fig. 1c. Panel (c) is a plain radiograph. Arrow shows a large bone erosion.

Unsuspected from physical examination and plain radiographs, DECT shows a large urate tophus at the supra-patella region of the left knee, the length exceeding that of the patella (Fig. 1a), and having intense FDG signal in PET (Fig. 1c). X-ray shows only a small erosion to which the tophus is attached (Fig. 2c). The conventional MRI view shows only the inferior part of the tophus (Fig. 2a). The T2 sequences show that it is extra-articular (not shown). Equally surprising, although the posterior view of the DECT shows only scattered urate signals, MRI and PET reveal that they are part of another massive tophus (Figs 1b and 2b). These features are not seen in the corresponding images of the right knee (not shown).

**Figure 3:**
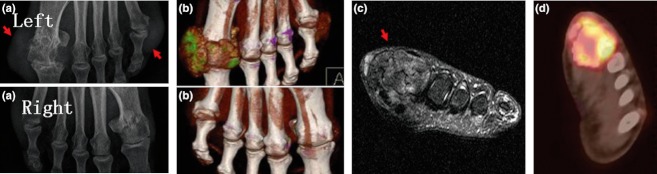
Images of the feet. Panel (a) are plain radiographs. Arrows indicate soft tissue swelling corresponding to the tophi in the three-dimensional reconstruction of the dual energy computed tomography (DECT) image shown in panel (b). In panel (b), gouty tophi are in green and brown colors. Panel (c) is an magnetic resonance coronal view. Arrow shows a mass corresponding to gout tophus around the first metatarsophalangial joint shown in the DECT image. Panel (d) is a positron emission tomography/computed tomography image. The yellow and red colors represent intense 2-fluoro-deoxy-d-glucose emission.

For the tophus in the first big toe, this is visualized as soft tissue swelling and bone erosion on X-ray (Fig. 3a, left foot panel), and urate as well as fibrous mass on DECT (Fig. 3b left foot panel), and a heterogeneous mass on MRI (Fig. 3c). PET again shows high FDG signal (Fig. 3d). For comparison, Fig. 3a,b shows that there is no significant tophus on the right foot.

There are several surprising discoveries in this study. The first is that DECT shows a huge tophus attached superiorly to the left patella, the size of the tophus exceeding that of the patella. This is completely unsuspected by physical examination and plain radiograph alone. The extent of this tophus extends beyond the MRI field. This is because conventional MRI covers only the areas of the knee joint which are required in assessment of other knee pathologies. Hence, our MRI is able to visualize only the inferior half of this particular tophus. Our MRI does show that this supra-patella mass has intensity in T1 and T2 sequences compatible with those of urate tophi. Although less spectacular, the images around the left big toe also confirm that physical examination and plain radiography have greatly under-assessed the size of the tophus at that location.

The second interesting finding in this study is that when we compare DECT to MRI, we notice that DECT does not necessarily provide all the information about the sizes of the tophi. MRI of the left knee reveals a large tophus situated in a posterior location. However, DECT in the same location reveals only mildly scattered urate compounds. Apparently, this tophus consists of more cellular or fibrous structures than urate crystals.

The third and final contribution in this paper is that, similar to two other papers, PET does show that gout tophi reveal significant inflammation. This is compatible with the histology of recently reported tophi.^7^ How inflammation prevents the crystals from being solubilized remains an enigma. Since urate solubility is highly dependent on the pH of the environment, perhaps the inflammation and associated fibrosis creates a pH of the surrounding tissues high enough to protect the crystals from being rapidly dissolved. New techniques of visualizing pH *in vivo* would be a useful direction in future research.^8^
